# Core Competencies for Chronic Disease Prevention Practice

**DOI:** 10.5888/pcd16.190101

**Published:** 2019-10-24

**Authors:** Mary Kane, Jennifer Royer-Barnett, Jeanne Alongi

**Affiliations:** 1 Concept Systems, Inc, Ithaca, New York; 2 National Association of Chronic Disease Directors, Decatur, Georgia

## Abstract

Chronic disease prevention practice is an important specialization within public health and health care that connects chronic conditions, causes, prevention tactics, and population-based health promotion modalities. Required competencies for successful chronic disease prevention and health promotion encompass leadership, epidemiology, program practice, and evaluation, among others. In 2007, the National Association of Chronic Disease Directors (NACDD) developed and codified the Core Chronic Disease Prevention Competencies (Competencies), a standard set of competencies for professionals in chronic disease prevention and control. NACDD also devised support tools to assist individuals and managers in increasing capacity and opportunities for member growth, thereby benefitting the agencies they serve. In revisiting the Competencies in 2015 through 2018, the NACDD Professional Development Committee reviewed uses, conducted member surveys, polled NACDD councils, and produced recommendations. The goal of this process was to recognize rapid changes in the environments, practices, and characteristics that affect chronic disease prevention, both at the population level and for individual groups at risk during the past 10 years. In addition, opportunities existed to benefit from the changes in technology that have increased demands on health professionals, who as a result have had to adapt to these changes. We worked with the NACDD Learning and Professional Development Committee and reviewed learning offerings, other related competency sets, and tools for performance assessment. The results of the review include a final set of Competencies and subcompetencies, a guide to using the competencies, and a fully integrated interactive assessment tool used by individuals, managers, and teams. Going forward, NACDD’s strategic focus includes a regular review of the Competencies and building chronic disease learning assets.

**Summary**

**What is already known on this topic?**

The National Association of Chronic Disease Directors developed the Core Chronic Disease Prevention Competencies (Competencies) in 2007 and created a competency-based tool kit.

**What is added by this report?**

The Competencies were updated from 2015 through 2018, which resulted in a redesign of the Competency-based tool kit. The updated tool kit included a digital, web-based self-assessment and team assessment tool for professionals and managers; a guide to using the Competencies; and templates for managers.

**What are the implications for public health practice?**

This article discusses the value of ensuring that sets of professional competencies and tools are current, relevant, and easily accessible to build workforce capacity and demonstrate ongoing commitment to learning and professional development in chronic disease program environments.

## Background

Chronic disease prevention practice is an important specialization within public health and health care. It intersects and connects the causes and prevention strategies associated with chronic conditions and the population-based health promotion modalities that affect the health and well-being of the population. Competencies for chronic disease prevention and health promotion encompass leadership, epidemiology, program practice, and evaluation, among others (1). Competencies are described here as the combination of observable and measurable knowledge, skills, abilities, and personal attributes that contribute to enhanced employee performance and support organizational success (2).

Many professions have an agreed-on set of knowledge, skills, aptitudes, and traits that are expected in their professions but not codified in a standard set of competencies and guidelines. This was the case for chronic disease prevention practice until the Core Chronic Disease Prevention Competencies (Competencies) were formalized in 2007 by the National Association of Chronic Disease Directors (NACDD) (1). NACDD established these Competencies through an iterative, member-driven process bounded by practice standards and peer-reviewed literature (1,3). NACDD’s mission is to improve the health of the public by strengthening state-based leadership and expertise for chronic disease prevention and control. The association’s initiatives support the work of state chronic disease units and their partners and build prevention and health promotion practice capacity at both the staff and organizational levels. NACDD’s membership includes over 6,500 professionals in 59 state and territorial health departments. NACDD used the Competencies to identify training and professional development needs for state chronic disease prevention and health promotion practice and to develop tools to support human resources decisions (eg, job description planner, interview guide, competency assessment tool). Additionally, NACDD created learning opportunities to build workforce capacity in the identified competencies areas including chronic disease academies, webinars, and chronic disease prevention leadership meetings. Data from the NACDD Survey of States indicated that 17 states conducted employee assessments by using this tool in 2017. No data are available about the overall ratings or applications of the results of use.

Rapid changes in environments, technology, practices, and science relevant to chronic disease prevention during the last decade required that state health departments develop flexible, responsible, and effective means and innovative approaches to chronic disease prevention and health promotion. At the same time, growth in evidence-based practice and organizational effectiveness science have resulted in an evolution of the skills, knowledge, and attributes used in chronic disease prevention practice. In 2015 in response to these contextual factors, NACDD leadership reviewed and revised the 2007 Competencies to make them more relevant and usable for learning, professional development, and agency needs. The objectives of this endeavor were to review and revise as necessary the 2007 Competencies for relevance to current and forecasted practice requirements; to identify and understand how these competencies and any revision may relate to relevant practice frameworks; and to begin the implementation and dissemination activities that would increase use of the Competencies. The resulting report and recommendations, entitled NACDD Core Chronic Disease Competencies: Updated June 2016 was finalized in 2017 (4).

## Revision Process for Competencies

This project was overseen by NACDD’s Professional Development Committee (PDC), an advisory group of 15 members from state health department chronic disease units who conducted regular conference calls and meetings. The review and revision process took place over 3 years. Building on the comprehensive and inclusive process taken to construct the 2007 Competencies (1), the Committee used a multimethod approach to validate and refine the Competencies and to develop dissemination and implementation recommendations. The Committee checked in repeatedly with key stakeholders and reacted to progress as findings were synthesized. This iterative process had 5 steps (Figure).

**Figure:**
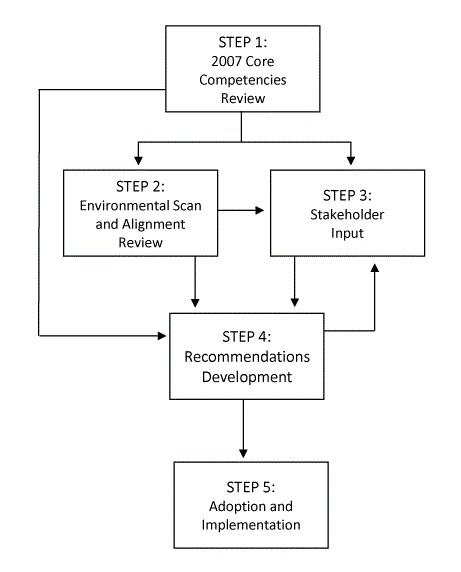
Iterative 5-step process for refining and implementing the Core Chronic Disease Prevention Competencies, 2015–2018.

**Step 1: 2007 Core Competencies review.** The PDC reviewed documents and publications related to the 2007 Competencies including the self-assessment tool, human resources support tools for job descriptions, and interview guides. The Committee sought, discussed, and reviewed relevant principles, terms, and concepts that were not included in the original version and identified gaps with current approaches to public health workforce development.

**Step 2: environmental scan and alignment review.** The environmental scan asked several members of NACDD leadership to identify frameworks and practice recommendations relevant to chronic disease prevention and health promotion practice. These were compared against the 2007 Core Competencies and were analyzed for gaps and alignment with draft recommendations as they were developed.

**Step 3: stakeholder input.** Stakeholder input was collected at multiple times throughout the project through surveys, interviews, and postwebinar polling. Inquiries focused on the relevance of Competencies to practice areas and needed skills, the relevance and utility of existing implementation tools, forecast of future needs, and identification of redundancy. Stakeholders were chronic disease prevention practitioners and subject matter experts including NACDD members, NACDD staff and consultants, and partners.

**Step 4: recommendations development.** As results were accumulated, revisions to the competency list and recommendations for tool development, implementation, and dissemination were drafted. This iterative process continued until the Committee agreed that the recommendations reflected a consensus based on the findings from steps 1 through 3.

**Step 5: adoption and implementation.** The NACDD board of directors formally adopted the Committee’s recommendations in January 2018 and implementation and dissemination activities began.

## Development of Revised Competencies

### Step 1: 2007 Core Competencies review

The PDC found that the 2007 Competencies did not emphasize key public health and chronic disease prevention aspects including health equity, cultural competence, and quality improvement. The Committee also forecasted gaps related to system-level and policy-level concepts in chronic disease such as social determinants of health, health disparities, payer for health care services, and communities’ relationship to chronic disease prevention and control.

### Step 2: environmental scan and alignment review

Six documents describing frameworks that were particularly relevant to chronic disease prevention practice in state health departments were found in the environmental scan. The subsequent environmental scan results were consistent with prioritizing evidence-based decision-making regarding public health programming, administrative practices, and organizational capacity development:

- **Administrative evidence-based practices**. This literature review proposed 5 priority administrative practice areas for fostering more effective public health. These were workforce development, leadership, organizational climate and culture, relationships and partnerships, and financial processes (5).
- **Chronic disease domains**. The National Center for Chronic Disease Prevention and Health Promotion of the Centers for Disease Control and Prevention (CDC) articulated a set of domains for chronic disease prevention practice around which state health departments can coordinate chronic disease prevention efforts across traditional categorical lines, thereby optimizing effectiveness and efficiency of public health practice. These domains are epidemiology and surveillance, environmental approaches, health care systems interventions, and community programs linked to clinical services (6).
- **Core competencies for public health professionals**. The Council on Linkages Between Academia and Public Health Practice identified a set of skills for general public health practice. This set is based on the 10 essential services and is broadly applicable to public health practice, education, and research (7,8).
- **Model for chronic disease coordination**. CDC’s National Center for Chronic Disease Prevention and Health Promotion proposed a model for building capacity for chronic disease prevention practice in state health departments that was based on a review of experience implementing the federally funded Coordinated Chronic Disease Program. Proposed model elements included evidence-based interventions, consistent communications, strategic use of staff, strong infrastructure, focused agenda, identification of functions, comprehensive planning, management resources, relationship building, and collaborative leadership (9).
- **Public Health Accreditation Board (PHAB) standards and measures**. PHAB developed standards for voluntary health department accreditation with the objective of promoting high performance, continuous improvement, and accountability. The 12 evidence-based domains are assessment, investigation, education, community engagement, policies and plans, enforcement, access to care, workforce competency, quality improvement, evidence-based practices, administration and management, and governance (10).
- **State Activation and Response (STAR)**. In response to requests from state chronic disease directors, NACDD developed an organizational capacity development model to assist states in strengthening their ability to successfully practice chronic disease prevention and health promotion. The STAR framework includes evidence-based public health practice, leadership, management and administration, organizational climate and culture, partnerships and relationships, and workforce development (11).

### Step 3: stakeholder input

With NACDD staff support, the PDC surveyed NACDD members who responded to an open invitation in July 2015, resulting in 74 responses. NACDD’s diabetes council provided collective feedback. Respondents provided information on location, type of organization, and number of years in chronic disease practice. NACDD members from 37 states and 2 US territories responded to the survey; most were from state health departments (71%, n = 49). The rest of the respondents were from local health departments (13%, n = 9) or worked at other organizations or agencies (16%, n = 11) (5 respondents skipped the question).

The range of work experience in chronic disease prevention was 0–3 years or more than 10 years; of the 70 respondents (4 respondents skipped the question), 38 had 10 or fewer years of experience, and 32 had more than 10 years of experience. To explore any potential themes related to length of work experience, the PDC compared the responses of practitioners with more than 10 years of experience with those who had 10 years or fewer. When the samples were cross-referenced, responses were similar and allowed the PDC to generalize across all levels of experience (4).

Survey findings, key informant interviews, and webinar polling confirmed the need for revision to the original competencies set and revamping the implementation and dissemination approach previously in place. Specifically, respondents cited the importance of competency areas such as health equity, health systems transformation, quality improvement, and design thinking, and they reflected on the priority of each competency area. As a result, the PDC reordered the subcompetencies under each competency area to begin with the highest priority and most essential items (6). The proposed Competencies were used in the development of the May 2017 general member webinar, which introduced NACDD members to the new Competencies and described plans to apply the Competencies in both standard and new ways to better serve members. In 2016 and 2017, 289 NACDD members attended or viewed 3 general member webinars, which reviewed the updated Competencies. Input on implementation and dissemination showed opportunities for technology-based assessment tools and ongoing training support regarding tool use.

### Step 4: recommendations development

As findings were collected in steps 1 through 3, they were synthesized by the PDC. Draft recommendations were circulated in several iterations and shared with key informants for confirmation until the Committee agreed they had reached consensus.

The resulting final recommendations expanded on the original 2007 Competencies framework. The report recommended that the competencies be relabeled “competency areas” rather than “domains” to avoid confusion referencing other related competency sets and to label the items themselves as “subcompetencies” rather than “competencies.” No new competency areas were added, indicating the utility of the 2007 framework (4).

Twenty-five new subcompetencies were added; 15 of those items are related specifically to health equity (Table 1). Cross-referencing these new subcompetencies led to an update to the self-assessment tool, consisting of 52 items.

**Table 1:** Subcompetencies Added by Chronic Disease Competency Area, Core Chronic Disease Prevention Competencies, National Association of Chronic Disease Directors, 2016

Subcompetency No.	Description	Goal
**Competency area 1: build support**		
1–19	Develop and support partnerships among public, nonprofit, and private entities to provide a comprehensive infrastructure to increase awareness, drive action, and ensure accountability in efforts to end health disparities and achieve health equity across the lifespan.	Health equity
**Competency area 2: design and evaluate programs**		
2–9	Apply and use scientifically sound evaluation techniques.	—
2–10	Present accurate demographic, statistical, programmatic, and scientific information effectively for professional and lay audiences.	—
2–11	Use and apply economic evaluation techniques.	—
2–12	Incorporate geomapping techniques into data analysis.	—
2–13	Report and communicate data effectively (visually and verbally).	—
2–14	Understand how to invest in community-based participatory research and evaluation of community-originated intervention strategies to build capacity at the local level for ending health disparities.	Health equity
2–15	Develop skills to expand and transfer knowledge generated by research and evaluation for decision-making about policies, programs, and grant-making related to health disparities and health equity.	Health equity
**Competency area 3: influence policies and systems change**		
3–8	Identify local government structures. Demonstrate skill in engaging local government, health discussions, planning, etc.	—
3–9	Clearly articulate the impact of social determinants of health policies on health (include nontraditional partners such as housing, transportation, community design, for example).	Health equity
3–10	Ensure the availability of data of all racial populations and transferring knowledge related to racial, ethnic, and underserved populations.	Health equity
**Competency area 4: lead strategically**		
4–14	Demonstrate ability to build capacity at all levels of decision-making to promote community solutions for ending health disparities.	Health equity
4–15	Demonstrate ability to improve coordination, collaboration, and opportunities for soliciting community input on funding priorities and involvement in research and services.	Health equity
4–16	Demonstrate ability to invest in young people to prepare them to be future leaders and practitioners to end health disparities.	Health equity
**Competency area 5: manage people**		
5–18	Demonstrate ability to develop and support the health workforce and related industry workforces to promote the availability of cultural and linguistic services, program development, and others.	Health equity
5–19	Demonstrate ability to increase diversity and competency of health workforce and related industries through recruitment, retention, and training of racially, ethnically, and culturally diverse individuals and through leadership action by health care organizations and systems.	Health equity
**Competency area 6: manage programs and resources**		
6–19	Develop and manage budgets that cross multiple award and funding cycles.	—
6–20	Apply project management principles.	—
6–21	Apply economic principles and concepts to program management.	—
6–22	Develop a diverse funding portfolio: federal and state, foundations, hospital community benefit, and university-obtained grant dollars.	—
6–23	Demonstrate ability to implement strategies to promote health equity and investing the resources to that end.	Health equity
6–24	Demonstrate ability to apply a health equity lens to the development, execution, and evaluation of programs.	Health equity
**Competency area 7: use public health science**		
7–21	Demonstrate a commitment to social justice and health equity.	Health equity
7–22	Integrate principles of social justice into public health practice and promotion.	Health equity
7–23	Demonstrate cultural sensitivity toward underserved populations.	Health equity

Abbreviation: — , not applicable.

### Step 5: adoption and implementation

The NACDD board of directors formally adopted the recommended updated competencies in January 2018, and NACDD began dissemination activities. The Competencies are included in the NACDD Core Chronic Disease Competencies: Updated June 2016 (4), and the National Association of Chronic Disease Directors Competencies for Chronic Disease Practitioners, October 2016 (12). The Guide to Understanding and Using the Chronic Disease Competencies (13) was developed for easy member implementation of the Competencies.

Concept Systems, Inc (CSI), and NACDD leadership used the Competencies to support NACDD’s increased commitment to professional development to pursue the following actions. First, to emphasize the priorities and activate member engagement in learning and professional development as an association priority, the PDC was reactivated as the Learning and Professional Development Committee (LPDC). Second, the LPDC formed 4 strategic working groups to focus on the recommendations from the report and address or complete each recommendation (Table 2). The recommendations included adopting the Competencies and developing a complete set of tools, as well as conducting a robust assets inventory to support member learning needs aligned with the Competencies. Third, CSI and NACDD leadership used NACDD’s previous assessment tools as the basis for a newly designed, technology-supported assessment tool available for continuous access (14). The assessment tool focuses on a subset of subcompetencies within the 7 chronic disease competency areas. First piloted at the September 2017 Chronic Disease Academy, the Chronic Disease Competencies Assessment Tool is now available for use in employee, manager, and team assessments within state and territorial chronic disease units. The tool is self-driven and provides explicit instructions for the user to provide ratings on each subcompetency in the self-assessment set, aggregate the ratings, score the ratings, and create an individual development plan that is a point-in-time blueprint. 

**Table 2:** Recommendations Accepted by the 2017 NACCD Board and Actions Taken to Address Each Item, Core Chronic Disease Prevention Competencies (Competencies), National Association of Chronic Disease Directors, 2015–2018

Recommendation	Action
**Revising competencies nomenclature**	For the Competencies, the “Seven Competency Domains” are now the “Seven Competency Areas” and statements originally called “competencies” are now called “subcompetencies.”
**Competencies order**	The order was changed based on survey respondents’ value rankings.
**Content additions**	Additional subcompetencies, including those focusing on health equity, were added, and language in other competencies was updated.
**Update of the competency assessment tools**	The Chronic Disease Competencies Assessment Tool was released in August 2018 and is available at https://tools.chronicdisease.org.
**Update of the inventory of NACDD LPD assets**	The LPDC formed a subgroup of members focusing on updating the inventory in time for the 2019 Chronic Disease Academy.
**Develop a lexicon of terms**	A glossary of terms from the Competencies is in development.
**Present the updated competencies in a newly designed package to promote use**	A guide with worksheets for individual, management, organization, and system planning based on the Chronic Disease Competencies has been developed and is under review by NACDD leadership and additions to the NACDD professional development web page are being developed by the LPDC.
**Increase promotion of the updated competencies**	Webinars and other LPD offerings now denote which chronic disease competency areas they address. Annual webinars on the competencies are offered.
**Encourage use of the competency framework**	The 2019 Chronic Disease Academy is being developed with the Competencies at the forefront.
**Require periodic review of competencies**	The LPDC has formed a subgroup of members focusing on developing a structured process to update the Competencies and completing the activity in 2019.
**Provide chronic disease staff with updated competencies for chronic disease practice**	The Competencies are available to all chronic disease staff on NACDD’s professional development web page: https://www.chronicdisease.org/page/Professional_Develop.

Abbreviations: LPD, learning and professional development; LPDC, Learning and Professional Development Committee; NACCD, National Association of Chronic Disease Directors.

The user and the manager both can revisit the plan and identify what progress has been made. The manager may also collect assessments from other team members and, via the capacity to aggregate across team members, conduct a team assessment to identify highlights, assess team progress, and notice gaps in the team’s capacity to address needs. This process supports the manager in making hiring and coaching decisions as the program’s needs change. The manager also has access to supporting tools in the tool kit. The job description builder and the interview planner are 2 tools that are included in the Assessment Tool. The data included can link to the Chronic Disease Competencies Assessment Tool to help in ongoing assessment. Further uses of the tool are described in the Guide to Understanding and Using the Chronic Disease Competencies (13).

## Implementation of Revised Competencies

The resulting Competencies set is consistent with other contemporary frameworks for effective public health practice and reflects the emerging need specific to public sector chronic disease prevention and health promotion roles. These updated Competencies offer a foundation for continued creation and delivery of professional development and learning opportunities that will allow state health departments to accelerate chronic disease prevention and improved population health outcomes. By reviewing related contemporary frameworks, we established the relationship of the NACDD Competencies to broaden the context in which competencies are established and used (12). In explicitly mapping the alignment between the updated competency set and frameworks such as the PHAB Standards (10), it is possible for NACDD and states to link professional development to develop and sustain public health accreditation status and other evidence-based organizational effectiveness paradigms.
